# Diagnosing Melanomas in Dermoscopy Images Using Deep Learning

**DOI:** 10.3390/diagnostics13101815

**Published:** 2023-05-22

**Authors:** Ghadah Alwakid, Walaa Gouda, Mamoona Humayun, N. Z Jhanjhi

**Affiliations:** 1 Department of Computer Science, College of Computer and Information Sciences, Jouf University, Sakakah 72341, Saudi Arabia; gnalwakid@ju.edu.sa; 2 Department of Electrical Engineering, Shoubra Faculty of Engineering, Benha University, Cairo 11672, Egypt; walaa.gouda@feng.bu.edu.eg; 3 Department of Information Systems, College of Computer and Information Sciences, Jouf University, Sakakah 72341, Saudi Arabia; 4 School of Computer Science (SCS), Taylor’s University, Subang Jaya 47500, Malaysia; noorzaman.jhanjhi@taylors.edu.my

**Keywords:** melanoma, deep learning, artificial intelligence, diagnostics, Inception-V3, InceptionResnet-V2

## Abstract

When it comes to skin tumors and cancers, melanoma ranks among the most prevalent and deadly. With the advancement of deep learning and computer vision, it is now possible to quickly and accurately determine whether or not a patient has malignancy. This is significant since a prompt identification greatly decreases the likelihood of a fatal outcome. Artificial intelligence has the potential to improve healthcare in many ways, including melanoma diagnosis. In a nutshell, this research employed an Inception-V3 and InceptionResnet-V2 strategy for melanoma recognition. The feature extraction layers that were previously frozen were fine-tuned after the newly added top layers were trained. This study used data from the HAM10000 dataset, which included an unrepresentative sample of seven different forms of skin cancer. To fix the discrepancy, we utilized data augmentation. The proposed models outperformed the results of the previous investigation with an effectiveness of 0.89 for Inception-V3 and 0.91 for InceptionResnet-V2.

## 1. Introduction

**Figure 1:**
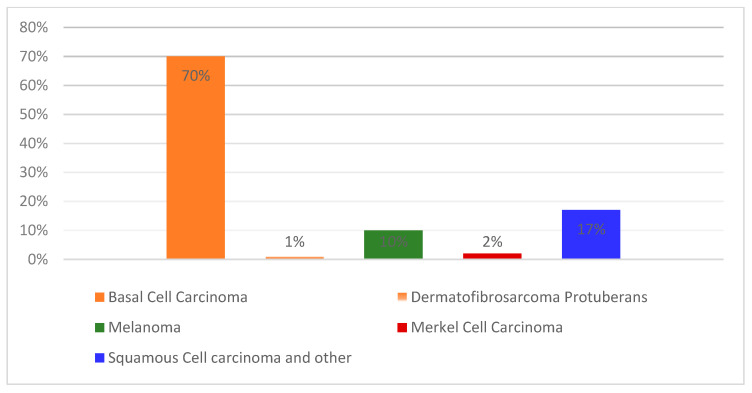
The prevalence of several forms of skin cancer (derived from Ref. [5]).

Among all cancers, skin tumors have the highest potential for malignancy [1,2]. Melanoma (MEL), basal cell carcinoma (BCC), squamous cell carcinoma (SCC), and non-melanoma comprise the most common types of skin cancer. However, actinic keratosis (AK), Kaposi sarcoma (KS), lymphoma, and keratoacanthoma [3,4] are exceptionally rare forms of cancer. Figure 1 depicts an upward trend in the incidence of all types of skin cancer.

The majority of cases of skin cancer fall into two categories: melanoma and non-melanoma [1,6,7]. Cancer mortality and medical expenditures are both increased by the presence of malignant lesions, so scientists have been working on techniques that can identify pre-malignant skin lesions with a high degree of accuracy and flexibility. Due to their rapid proliferation, invasion, and dissemination, malignant melanocyte cells must be identified at an early stage [8]. Dermoscopy is commonly used by specialists to identify the malignancy or benignity of a skin lesion.

The term “dermoscopy” refers to a method that makes use of a magnifying lens and a light source to enhance the visibility of various medical features [9,10]. It reveals the hidden morphologies to the naked eye. The ABCD rule [11], a seven-point checklist [12], and pattern analysis [13] are just some of the methods that have been established to enhance the precision of skin melanoma identification. However, dermoscopy images taken by laypeople have a prognostic validity of 75% to 84% for melanoma; the processing is time-consuming and subjective, depending on the dermatologist’s skill [14]. Professionals have created computer-aided diagnosis (CAD) methods to help them work around these problems [10,14]. Advances in computer-aided cancer diagnosis can be largely attributed to deep learning (DL)-based AI [15,16].

Applying DL methods in skin lesion classification helps automate the screening and early diagnosis of skin cancer, even in areas without easy access to dermatologists or laboratories [17]. Traditional classifiers [18,19] require feature extraction performed by humans before being supplied into computer-aided dermoscopy image processing.

In this research, various models were used (including InceptionV3 and Inception-Resnet). InceptionResnet-V2 incorporates the residual connections within the Inception design. For this reason, Inception-Resnet is the most reliable option. Computational effectiveness is poor and fewer parameters are realized in the Inception-Resnet model. Additionally, it provides a high-performance boost, efficient use of computing resources, and a slightly higher computation load, all of which contribute to the Inception-Resnet network’s high-performance output. 

Our examination of deep neural network (DNN) classification performance on the HAM10000 dataset led us to the conclusion that the employed InceptionResnet-V2 model was more accurate at classification than competing DNNs. This means that individual investigations may be needed to determine which network works best with specific medical imaging datasets.

As a consequence, the following are the paper’s primary contributions:

1. An enhanced super-resolution generative adversarial network (ESRGAN) was employed using 10,000 training photos to generate high-quality images for the Human against Machine dataset (HAM10000 dataset [20]).
2. Because the HAM10000 dataset contains uneven data, we employed augmentation to balance the data throughout all classes.
3. A deep comparative evaluation employing several assessment parameters such as accuracy, specificity, sensitivity, a confusion matrix, and the F1-score established whether the proposed system was feasible.
4. Together with Inception-V3 and InceptionResnet-V2, it was used to fine-tune the weights of HAM10000-trained networks.
5. To boost the recommended method’s scalability and safeguard against overfitting, we used a supplementary training procedure assisted by several training strategy variations (e.g., data augmentation, learning rate, batch size, and validation patience).

For the identification of numerous skin lesions, this research proposed an optimization technique involving transfer learning models. For this, we used Inception-V3 and InceptionResNet-V2 architectures to pre-train the weights of each model. The HAM10000 dataset, which contains images of skin lesions, was utilized to compare the models’ results. The dataset’s class imbalance necessitated an oversampling technique. The rest of the paper is organized as follows. Section 2 displays the cited references. In Section 3, we detail the dataset, the analysis methods employed, and the proposed methodology. The findings of the experiments are presented in Section 4, and their analysis follows in Section 5. In Section 6, we provide a summary.

## 2. Related Work

Commonplace machine learning (ML) and DL techniques have been employed in CAD systems to process images of skin lesions following the standard image analysis pipeline. Several methods and approaches have been tried for picture pre-processing, image segmentation, feature extraction, and classification, but none have yielded satisfactory results [21,22]. The low accuracy rate of the classical classifiers, the high sophistication of segmenting the region of interest, and the need for specialized knowledge to obtain beneficial properties associated with the physical characteristics of skin lesions all contribute to the present perspectives’ restricted abilities. Because of these limitations, CAD systems still require human input. 

For instance, Albahar [23] improved a DL model for malignant melanoma detection utilizing 145 dermatologists from 12 German hospitals to make final diagnoses. Tembhurne et al. [24] presented a fresh solution to address the issue of skin cancer diagnosis by merging ML and DL methods. The DL model extracted features from images using sophisticated neural networks (NNs), while the ML model used techniques to analyze those features. Mazhar et al. [25] addressed the fundamental steps necessary to develop melanoma diagnosis software, which centered on two aspects: images with full segmentation and DL-based skin lesion tracking. Haenssle et al. [26] compared 58 dermatologists’ diagnoses to a Google Inception V4 DL model. The data included 100 dermoscopic and digitalized patient photos and medical documents. Furthermore, Alenezi et al. [27] suggested using dermoscopic images of skin lesions as part of an ongoing framework for identifying melanomas. In order to better examine dermoscopic images without being distracted by hair, that model developed a workable pre-processing strategy based on layer-by-layer dilation and pooling. The processed photos’ features were then extracted using a deep residual NN.

Another approach presented by Inthiyaz et al. [28] provided a dependable real-time pedagogical resource for medical students utilizing computational methods for image analysis, processing, and classification while taking into account a wide variety of image properties. A diagnostic analysis was produced after images were cleaned of unwanted noise and adjusted to improve their overall clarity. Finally, features were derived from the image using NN, and the images were classified using the softmax classifier method.

As an unresolved problem, optimization and fine-tuning can be incorporated into transfer learning’s already-established base model configuration. Table 1 highlights the several DL approaches [17,21,26,27,28,29,30,31,32] used to spot skin irregularities in photographs. The study’s findings suggested that there are many unanswered questions regarding how to identify and diagnose melanoma. Therefore, new models may enhance the efficiency of melanoma diagnosis.

## 3. Proposed Methodology

**Figure 2:**
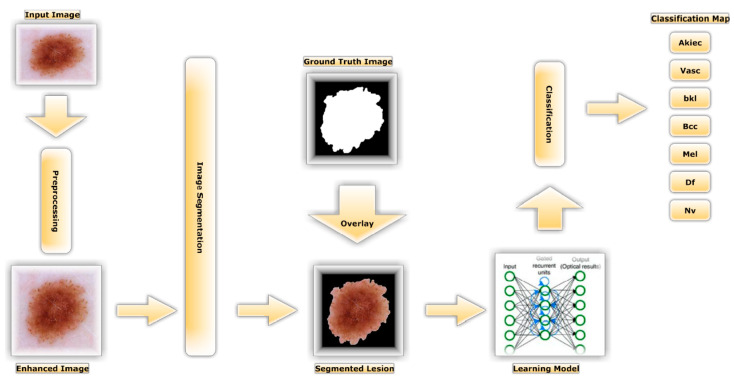
Proposed Approach for Identifying Skin Cancer.

Throughout this section, we go into painstaking depth regarding our methodology. As illustrated in Figure 2, there were three primary steps to this method. Pre-processing skin photos improved their quality for categorization by removing minute noise. The skin image’s edges were then delineated using ground truth, and the backdrop was then removed. Then, we applied many distinct segmented images in the transfer learning models. Automated learning of informative representations from skin images was accomplished using transfer learning models. In the following sections, we will outline the details of the proposed method.

### 3.1. HAM10000 Dataset

**Figure 3:**
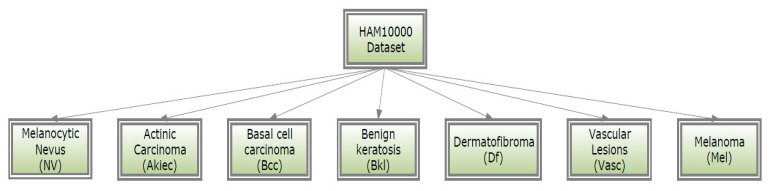
Classes of HAM10000 dataset.

The identification performance of the proposed models was assessed using the HAM10000 [20] dataset of lesions with pigmentation. The authors compiled dermatoscopic images from various demographics that were captured and stored employing various techniques. As a training set for scholarly use, it was anticipated to contain 10015 dermatoscopic images divided among seven classes, as seen in Figure 3. 

Histopathology was employed to verify the diagnosis in over 50 percent of the cases; in the other half, the truth was established through a subsequent examination and the agreement of experts. The HAM10000 metadata file contains a lesion-id column that may be utilized to track the position of the lesion across the many photos that comprise the dataset.

The HAM10000 dataset was separated into training, validation, and testing data as illustrated in Table 2, where 327 were Akiec, 514 were Bcc, 1113 were Mel, 142 were Vasc, 6705 were Nv, 115 were Df, and 1099 were bkl. 

### 3.2. Image Pre-Processing Step

Maximizing dermoscopy image resolution and removing various types of noise from images of skin lesions were also essential steps in bringing the proposed strategy into practice. A high-quality image is essential for developing a trustworthy model for the classification of skin lesions. Image segmentation, enhancement, data supplementation, resizing, and normalization are all a part of this process. Overfitting occurs when there are more variables to learn than can reasonably be accounted for due to an increase in network complexity in the model. Overfitting due to a small and uneven sample size of training photos was addressed by splitting the HAM10000 dataset into three distinct parts (e.g., training, verification, and assessment), and then data augmentation was employed to even out the forecasting capability across the board. Masks for rotation, reflection, shifting, and scaling are provided alongside the enhanced images for each image in the dataset.

### 3.3. ESRGAN

**Figure 4:**
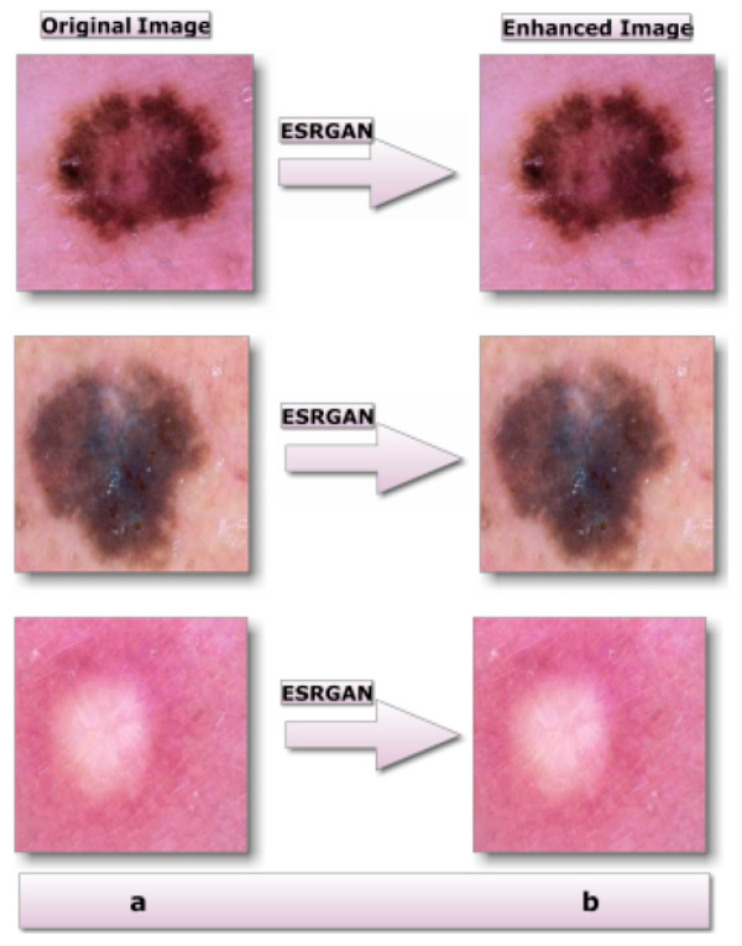
Results of the suggested image-enhancement technology: (**a**) unprocessed image; (**b**) processed image.

Ledig et al. [33] developed an Enhanced Super-Resolution Generative Adversarial Network (ESRGAN). By training generators and discriminators against each other, such technique, which is based on adversarial learning, generates texture features that are compatible with the distribution of genuine images. By extracting visual features at a single scale, this method achieves super-resolution. An image super-resolution rebuilding may be employed to determine the corresponding image of high quality from one or more photographs of different resolutions as shown in Figure 4. Examples of effective applications of this technology include remote sensing, medical imaging, picture compression, video monitoring, and military applications. Many academics have focused on image super-resolution since it is an important image-processing technology, and there have been numerous effective ways of achieving image super-resolution proposed [33].

### 3.4. Segmentation

**Figure 5:**
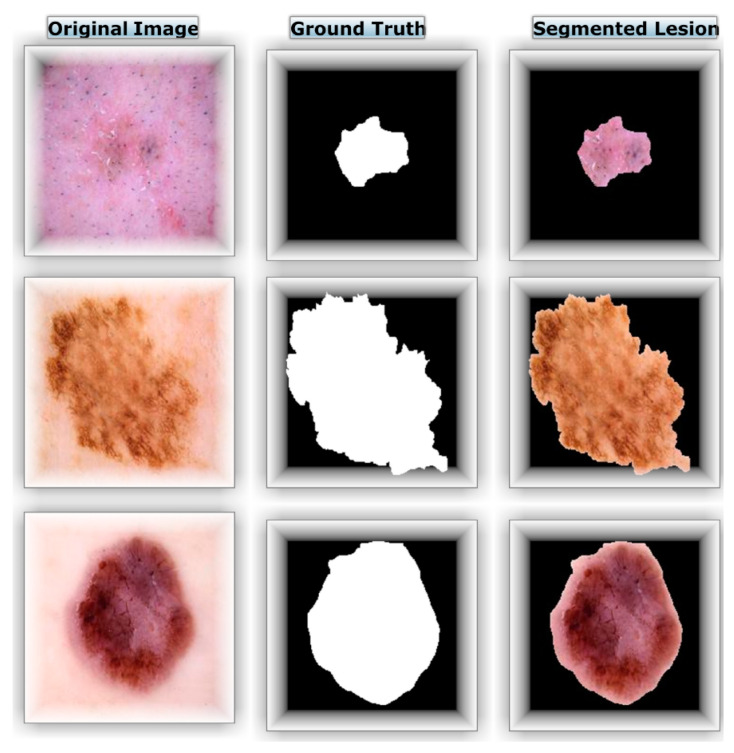
Main image, ground truth, and segmented ROI examples.

The dermoscopy images were processed according to the image preparation technique in order to extract the ROI. The original images were multiplied by the images that reflected their respective ground truths in order to create the ROIs displayed in Figure 5.

### 3.5. Data Augmentation

**Figure 6:**
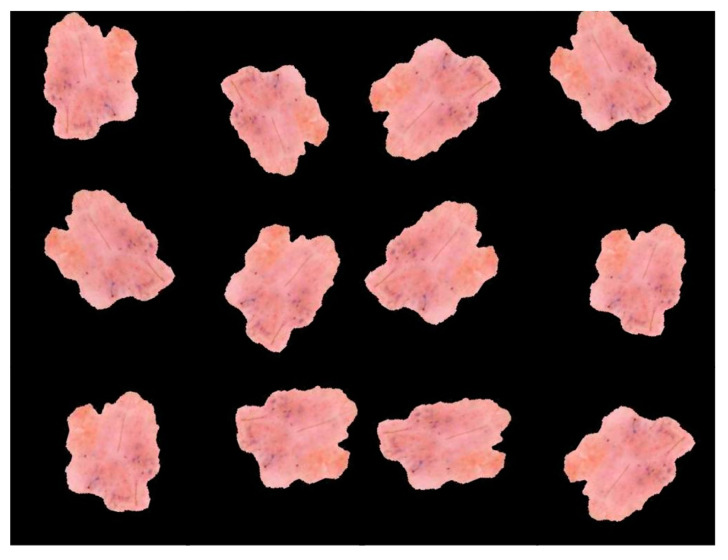
Image augmentation examples for the exact same image.

Prior to actually presenting the DNN with the original dataset images, we added more data to the training set as a preliminary step. Data augmentation is the most common way to avoid overfitting when training models. This is done by adding new images to the dataset in a way that retains the class information [8]. The main idea is that changes that can be made to replicable data do not change what the image means, so new samples can be made. Most of the time, increasing the amount of training data for DL models improves their performance. When it comes to dermatological images, we can make a lot of adjustments to each one by using what makes it distinctive. For these images, fading, flipping either vertically or horizontally, or spinning the images by a specific angle does not affect the performance of deep NNs. As ways to improve the data, horizontal flips; random rotations between 90 and 270 degrees; and changes to the saturation, exposure, and hue values of the original images were chosen. In particular, these parameters were set to have values of 1.5, 1.5, and 0.1, respectively. Each image in the training set was subjected to the changes listed above, giving the network a new sample. In this study, we used the ImageDataGenerator interface of Tensorflow2.0 to flip, rotate, and shift the input image data. Adding significantly modified versions of existing data or new synthetic data derived from existing data is how data augmentation works to increase the total amount of available data, as seen in Figure 6. 

It was crucial that the DL models be trained on a large number of photos that were fairly distributed because there was a noticeable disparity in the dataset (see Table 2). As demonstrated in Table 3, if we followed this reasoning and applied augmentation (oversampling) processes to the relevant classes after the dataset had been balanced, we obtained well-balanced data with about equal numbers of images in each category.

### 3.6. Transfer Models for Learning

To solve a new problem that is similar to the original problem but unique, ML practitioners can engage in a process known as transfer learning. In comparison to the conventional neural network, it simply requires a small amount of training data and a quick training period to attain a high accuracy. A discussion of the transfer models employed is presented in the following subsections. 

### 3.7. Model Training Utilizing Inception-V3

The theoretical underpinnings of the method are laid out and described in this subsection. Among these transfer learning pre-trained deep models is Inception-v3 [11,12], which builds on the design of its predecessors Inception-v1 [34,35] and Inception-v2 [36,37]. ImageNet datasets [38,39] have been used to train the Inception-v3 model, which then has been utilized to recognize a hundred distinct classes. Error rates have dropped from 17.3% for the best system to 3.5% for the top five in ImageNet.

In particular, the technique developed by Serre et al. [40]—which may involve multiple levels of processing—served as an inspiration for Inception. Using the method proposed by Lin et al. [41], the creators of Inception increased the accuracy of the neural networks used in the system. They were immune to computational limitations because the dimensions had been shrunk to 11 convolutions. Using Inception [42], scholars have significantly reduced the period and effort needed for DL picture classification. They aimed to strike a compromise between the two commonly utilized techniques for boosting performance; namely, expanding in depth and width and separating data into distinct layers without resorting to any empirical evidence or empirical analysis. The Inception DL system’s 22-layer architecture, in which every filter is a learned one, was designed with this specific end in mind. Input into the next layer was generated from highly correlated categories using a correlation statistical analysis based on the work of Arora et al. [43]. Eventually, after dimension reduction, every one of these layers is reduced to a series of 1x1 convolutions [40].

### 3.8. Model Training Utilizing Inception-Resnet

State-of-the-art performance was achieved in the 2015 ILSVRC challenge by including residual connections in a more conventional architecture; the resulting network was competitive with the most recent generation of the Inception-V3 network. In light of this, one may reasonably speculate as to whether combining the Inception architecture with residual blocks might yield any beneficial results. Regarding Inception network training using residual links, the training time for InceptioResnet-V2 [43] was proven to be drastically reduced. Even though Inception networks with residual connections are more expensive, there is some evidence that they outperform those with no residual connections. Szegedy et al. [44] introduced various novel, simplified topologies for Inception networks (both residual and non-residual). The ILSVRC 2012 classification challenge benefited greatly from these improvements when applied to single-frame identification.

## 4. Experiments and Results

In this section, we begin by giving an overview of the experimental setup by describing the models and parameters used, the datasets we utilized, and the hardware specifications of the machines used to conduct the experiments. The outcomes for each dataset and model comparison follow.

### 4.1. Instruction and Deployment of Inception-v3 and Inception-Resnet-V2

Transfer learning models were tested on the HAM10000 dataset and evaluated by comparing them to the best practices; 90% of the data (9016 photos) was for training and 10% was for testing (984 images). The validation procedure employed 10% of the training set (992). Each image was reduced to 227 by 227 by 3 pixels and magnified to a size of 39,430 throughout the training phase. An RTX3060-equipped Linux PC with 8 GB of RAM was used to test the TensorFlow Keras. Eighty percent of all TL models were trained on an uninformed collection of photos. After training, a validation set comprising 10% of the data was used to ensure that only the most accurate weight combinations were kept. Approved models were pre-trained on the HAM10000 dataset using the Adam optimizer and a learning rate approach that reduced the learning rate while the model was inactive for the validation patience. The following hyperparameters were passed to Adam during training: 0.90 momentum, 10 patience, and 50 epochs. The range of batch sizes ranged from 2 to 64, and each increment was double the preceding value. As part of our arsenal of methods to stop the spread of infectious forms, we also used a method called “batching”.

### 4.2. Criteria for Assessment

Throughout this subsection, we describe the study’s evaluation measures and the outcomes. The classifier effectiveness is a widely used measure of classification performance (Ac). Equation (1) depicts the formula, which was calculated by dividing the total number of examples by the proportion of correct identifications. Typically, the performance of image-classification systems is measured by their sensitivity and specificity. Equation (2) presents a formula for specificity that becomes more accurate as more photos are accurately labeled. The number of pictures in the dataset that had a linear relationship was determined according to Equation (3). More accurate predictions can be expected from a system with a higher F-score. Accuracy and sensitivity are not enough to determine a system’s value. The formula for determining the F-score (Fsc) is stated in Equation (4). The top N accuracy was the fourth measurement, and it refers to how well the model N’s highest likelihood responses fit the anticipated softmax distribution. If one of the N predictions was the correct label, then the classification was valid.

(1) $$Ac=\frac{T^{p}+T^{n}}{T^{p}+T^{n}+F^{p\;}+F^{n}}$$

(2) $$Specificity=\frac{T^{n}}{T^{n}+F^{p\;}}$$

(3) $$Sensitivity=\frac{T^{p}}{T^{p}+F^{n\;}}$$

(4) $$Fsc=2*\left(\frac{Pre*Rec}{Pre+Rec}\right)$$

The abbreviation T^p^ indicates “true positive”, T^n^ indicates a true negative, F^p^ indicates a false positive, and F^n^ indicates a false negative.

### 4.3. Effectiveness among Several DNN Models

**Figure 7:**
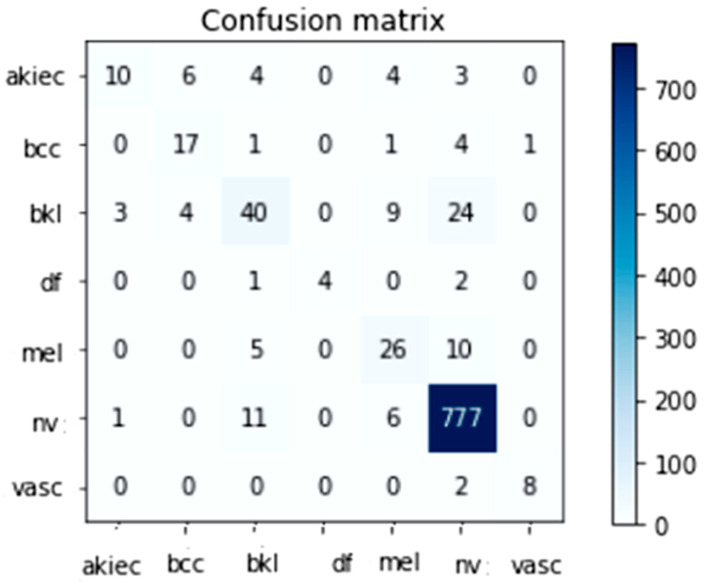
The Inception-V3 Confusion Matrix Top Performer.

**Figure 8:**
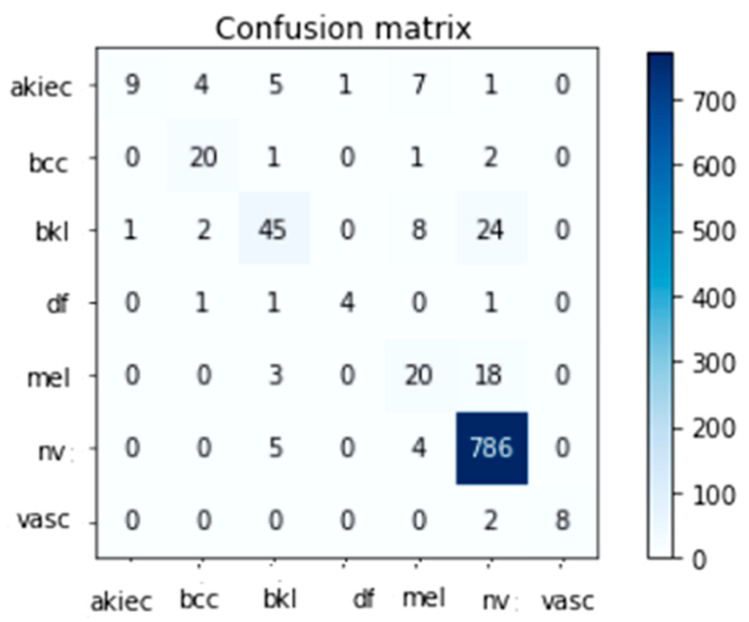
The InceptionResnet-V2 Confusion Matrix Top Performer.

Several TL classification methods were trained and validated using the HAM10000 skin lesion identification challenge dataset (including Inception-V3 and InceptionResnet-V2). The HAM10000 dataset was evaluated numerous times using a 90/10 crossover among training and testing, and the findings are given below. This split was decided upon to reduce the overall time required to finish the job. Classifiers were trained with a batch size between 2 and 64 and learning rates of 1E^4, 1E^5, and 1E^6 for Inception-V3 and InceptionResnet-V2. Freezing different numbers of layers allowed Inception-V3 and InceptionResnet-V2 to be fine-tuned for optimal accuracy. An ensemble of models was generated by repeatedly running the same model with the same set of parameters. The accuracy varied from run to run because the weights were generated randomly. For Inception-V3 and InceptionResnet-V2 training on the HAM10000 dataset, only the maximum run results were maintained; these are reported in Table 4 and Table 5, respectively. The tables demonstrate that the highest success rates for Inception-V3 and InceptionResnet-V2 were 89.7% and 90.1%, respectively. The confusion matrices created by Inception-V3 and InceptionResnet-V2 are depicted in Figure 7 and Figure 8, respectively.

Table 6 and Table 7 indicate the total amount of test photos for every class in the HAM10000 dataset. With 95% specificity, 98% sensitivity, and 96% Fsc when using the Inception-V3 learning model and 94% specificity, 99% sensitivity, and 97% Fsc when using the modified InceptionResnet-V2 learning model, it was clear that the Nv class had the most data points out of all the classes tested (795 total).

It was demonstrated that dermatologists can benefit from using lesion photos to improve the accuracy of infection diagnoses and decrease their burden. 

### 4.4. Comparison to Alternative Approaches

Comparisons of effectiveness with respect to competing methods are shown in Table 8. According to Table 8, our method was more productive and efficient than competing methods. Overall, the accuracy rate of the suggested InceptionResnet-V2 model was higher than that of state-of-the-art methods by 91.26%. ESRGAN’s greater overall resolution constituted the main reason for the improvement’s success.

## 5. Discussion

The results of our analysis demonstrated that the alternative approaches were significantly less accurate. We ascribe the enhancement to a trifecta of factors, one of which was ESRGAN’s higher overall resolution. Moreover, we employed a variety of architectures, some of which were more effective than others at generalizing and adapting to various types of data. Medical image categorization could not be enhanced by transfer learning architectures due to the lack of distinguishing features. While Inception-V3 excelled at image detection, it underperformed the proposed InceptionResnet-V2 when applied to medical images. Due to their lack of semantic meaning for real images, InceptionResnet-V2 features are more flexible and generally applicable in medical imaging (compared to Inception-V2). As a result, fine-tuning enhanced the precision of the two models. Deep networks were found to be superior to their shallow counterparts when it came to distinguishing crucial elements when trained on a smaller sample. The successful outcomes of these methods are shown in Figure 7 and Figure 8.

## 6. Conclusions

It is now possible to diagnose seven different types of cancer quickly and accurately through the study of skin blemishes. To improve the contrast of the lesion image and get rid of noise, the proposed method made use of image-enhancing techniques. To avoid overfitting and increase the capabilities of the suggested DL approaches, Inception-V3 and InceptionResnet-V2 were trained on the forefront of pre-processed lesions using augmentation procedures. The presented approach was evaluated by employing images of lesions from the HAM10000 dataset. It was speculated that the Inception-V3 and InceptionResnet-V2 versions of the conception model had accuracy rates similar to those of dermatology specialists with board certification. The study’s originality and novelty also reside in the fact that it used ESRGAN as a pre-processing step for the numerous models it employed (Inception-V3 and InceptionResnet-V2). Our freshly trained model performed as well as, if not better than, the baseline. Tests comparing the suggested system to others showed that it outperformed the competition with 91.26% accuracy. The technique’s viability can only be established by putting it through its paces on a huge dataset basically including a sizable number of future cancer predictions. DenseNet, VGG, and AlexNet are three techniques that show promise for future analysis of the cancer dataset.

## Figures and Tables

**Table 1:** Comparison of existing melanoma diagnostic methods.

Work	Dataset	Used Techniques	Number of Testing Images	Number of Training Images	Number of Classes
D. Mendes and N. da Silva [29]	University Medical Center Groningen Dermofit Atlas	ResNet-152	956	3797	12
S. Aijaz [30]	BFL NTU + Dermnet	Segmentation + VGG-19 + LSTM	188	1468	6
R. Zare and A. Pourkazemi [32]	HAM10000	U-Net (segmentation) + DenseNet121 (classification)	-	-	7

**Table 2:** Inadequate sample size prior to using augmentation techniques.

Class	Number of Images in Each Partition			
	Training Set	Validation Set	Testing Set	Total Set
Akiec	273	27	27	327
Bcc	451	31	32	514
Mel	1030	42	41	1113
Vasc	119	12	11	142
Nv	5115	795	795	6705
Df	101	7	7	115
Bkl	940	79	80	1099
Total	8029	993	993	10,015

**Table 3:** Equitable dataset using augmentation (oversampling) with segmented images.

Class	Number of Training Images
Akiec	5684
Bcc	5668
Mel	5886
Vasc	5570
Nv	5979
Df	4747
Bkl	5896
All Classes	39,430

**Table 4:** Highest Effectiveness via Finest Tuning with Inception-V3.

Acc	Top-2 Accuracy	Top-3 Accuracy	Specificity	Sensitivity	Fsc
0.897	0.960	0.981	0.89	0.90	0.89

**Table 5:** Highest Effectiveness via Finest Tuning with InceptionResnet-V2.

Acc	Top-2 Accuracy	Top-3 Accuracy	Specificity	Sensitivity	Fsc
0.913	0.968	0.986	0.90	0.91	0.91

**Table 6:** Detailed findings produced for every category by the Inception-V3 learning model.

	Specificity	Sensitivity	Fsc	Total Images
Akiec	0.71	0.37	0.49	27
Bcc	0.63	0.71	0.67	24
Bkl	0.66	0.5	0.57	80
Df	1	0.57	0.73	7
Mel	0.57	0.63	0.6	41
Nv	0.95	0.98	0.96	795
Vasc	0.89	0.8	0.84	10
Average	0.89	0.9	0.89	984

**Table 7:** Detailed findings produced for every category by the InceptionResnet-V2 learning model.

	Specificity	Sensitivity	Fsc	Total Images
Akiec	0.9	0.33	0.49	27
Bcc	0.74	0.83	0.78	24
Bkl	0.75	0.56	0.64	80
Df	0.8	0.57	0.67	7
Mel	0.5	0.49	0.49	41
Nv	0.94	0.99	0.97	795
Vasc	1	0.8	0.89	10
Average	0.9	0.91	0.9	984

**Table 8:** Comparison with other methods.

Reference	Model	Dataset	Acc
[45]	AlexNet	HAM10000	84%
[46]	MobileNet	HAM10000	83.9%
[47]	MobileNet, VGG-16	HAM10000	80.61%
[48]	SVM	HAM10000	74.75%
[49]	ResNet	HAM10000	78%
	Xception		82%
	DenseNet		82%
[50]	CNN	HAM10000	77%
[51]	MobileNet and LSTM	HAM10000	85%
[16]	RegNetY-3.2GF	HAM10000	85.8%
[52]	Inception V3	ISIC 2019	91%
Proposed	Inception-V3	HAM10000	89.73%
	InceptionResnet-V2	HAM10000	91.26%

## Data Availability

Will be furnished on request.
